# 2204. Excess Antibiotic Therapy for 5 Common Conditions in Primary Care: Low-Hanging Fruit for Outpatient Antibiotic Stewardship

**DOI:** 10.1093/ofid/ofad500.1826

**Published:** 2023-11-27

**Authors:** Payal K Patel, Whitney Buckel, Rachel Fletcher, Nick Gangwer, Jeni Hansen, Valoree K Stanfield, Adam Hersh, Eddie Stenehjem, Allan M Seibert

**Affiliations:** Intermountain Health, Salt Lake City, Utah; Intermountain Health, Salt Lake City, Utah; Intermountain Health, Salt Lake City, Utah; Intermountain Health, Salt Lake City, Utah; Intermountain Health, Salt Lake City, Utah; Intermountain Healthcare, Murray, Utah; University of Utah, Salt Lake City, UT; Division of Infectious Diseases, University of Colorado, Aurora, Colorado; Intermountain Healthcare, Murray, Utah

## Abstract

**Background:**

Improving duration can be a key intervention in outpatient antibiotic stewardship as most conditions require only 5 days of therapy or less. Cellulitis, acute otitis media (AOM), sinusitis, community-acquired pneumonia (CAP), and uncomplicated urinary tract infections (UTI) encounters account for the majority of outpatient antibiotic prescriptions and have been associated with durations longer than necessary. We characterized the duration of antibiotic prescriptions associated with these conditions in our primary care (PC) network.

**Methods:**

We retrospectively evaluated prescribing practices for cellulitis, AOM, sinusitis, CAP, and UTI encounters among patients ≥ 3 years-old from January 1^st^, 2022 – December 31^st^, 2022 in the Intermountain Health (IH) PC network. IH is an integrated healthcare system operating 119 PC clinics (69 family medicine, 22 internal medicine, 23 pediatric). System-specific evidence-based ambulatory guidelines for the conditions of interest during the study recommended 5-7 days of therapy. Encounters, patient characteristics, and prescription information were electronically extracted from the electronic health record. We analyzed duration of therapy by categories (≤5 days, 6-9 days, 10-14 days, or 14+ days) for each condition in pediatric (≥ 3-18 years-old) and adult (≥ 19 years-old) patients.

**Results:**

There were 14,918 and 38,790 encounters for the studied conditions among pediatric and adult patients, respectively. The mean age of pediatric patients was 9.0 years and among adults, 56.8. Pediatric and adult patients were predominantly White, non-Hispanic, female, and preferred speaking English (Table 1). Overall, only 10.4% (8,965) of all prescriptions were ≤ 5 days and consistent with the recommended duration. Duration of prescriptions associated with each condition are presented in Figure 1. UTI exhibited the highest percentage of ≤ 5 day prescriptions (22.1% pediatrics, 29.1% adults) while sinusitis had the lowest (0.9% pediatrics, 2.6% adults).

**Table 1.**
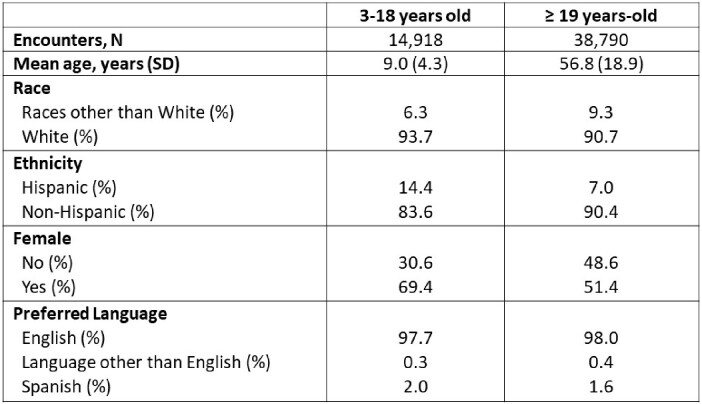
Total encounters for all studied conditions and patient characteristics

**Figure 1.**
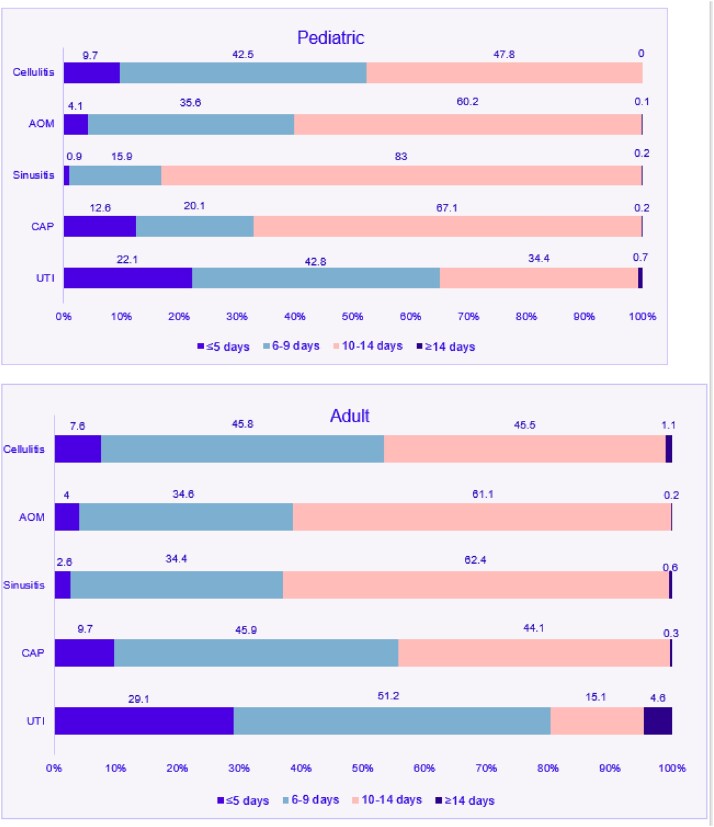
Duration of antibiotic prescriptions associated with cellulitis, acute otitis media (AOM), sinusitis, community-acquired pneumonia (CAP), and uncomplicated urinary tract infection (UTI) encounters for patients 3-18 years-old (pediatric) and ≥19 years-old (adult) in the Intermountain Health primary care network.

**Conclusion:**

Duration of therapy for cellulitis, AOM, sinusitis, CAP, and UTIs is almost always longer than the recommended 5 days of therapy in our PC network. There is substantial opportunity to improve duration for these conditions.

**Disclosures:**

**Payal K. Patel, MD MPH**, qiagen: Honoraria

